# Pulmonary Actinomycosis Mimicking Pulmonary Tuberculosis: A Diagnostic Challenge

**DOI:** 10.7759/cureus.102871

**Published:** 2026-02-03

**Authors:** Srikant K Malegaonkar

**Affiliations:** 1 Pulmonary Medicine, All India Institute of Medical Sciences, Nagpur, Nagpur, IND

**Keywords:** actinomyces israelii, anaerobic infection, lung cavity, pulmonary actinomycosis, tuberculosis mimic

## Abstract

Pulmonary actinomycosis is a rare chronic infection that can closely mimic pulmonary tuberculosis (PTB) in both clinical presentation and radiological appearance, particularly in tuberculosis-endemic regions. Delayed or missed diagnosis may result in prolonged inappropriate therapy and disease progression.

We report a case of pulmonary actinomycosis in a diabetic patient initially treated empirically for pulmonary tuberculosis, highlighting the importance of histopathological examination and anaerobic culture in patients with poor response to anti-tubercular therapy.

## Introduction

Pulmonary tuberculosis (PTB) is among the most frequent causes of prolonged fever, cough, and lung consolidation in India [1]. Anti-tubercular treatment (ATT) forms the backbone of PTB therapy. An inadequate response to ATT typically prompts evaluation for drug resistance, treatment non-compliance, or secondary infection. Pulmonary actinomycosis is a rare chronic infection caused by gram-positive filamentous anaerobic bacilli belonging to the family Actinomycetaceae. Poor oral hygiene, diabetes, aspiration, and chronic lung disease are important risk factors for acquiring pulmonary actinomycosis. Pulmonary actinomycosis is a challenging diagnosis to make as it closely mimics PTB in clinical and radiological presentation [2]. Histopathology and anaerobic cultures, typically not a part of routine testing, are crucial for diagnosis and treatment commencement [3]. We present a case illustrating this rare infection, highlighting salient points of management.

## Case presentation

**Figure 1:**
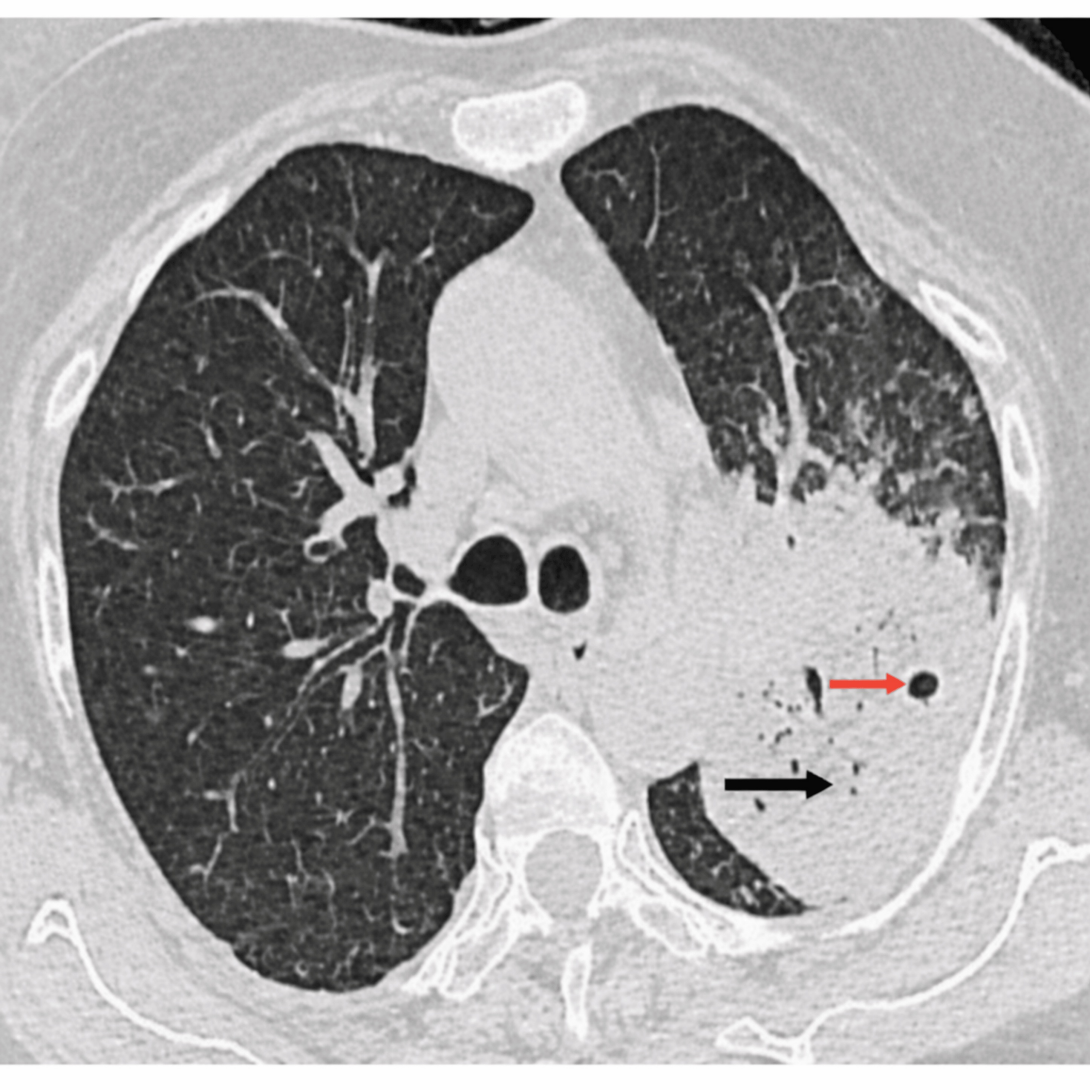
High-resolution computed tomography of the chest showing consolidation (black arrow) with cavitation (red arrow) involving the left upper lobe posterior segment and lingula

**Figure 2:**
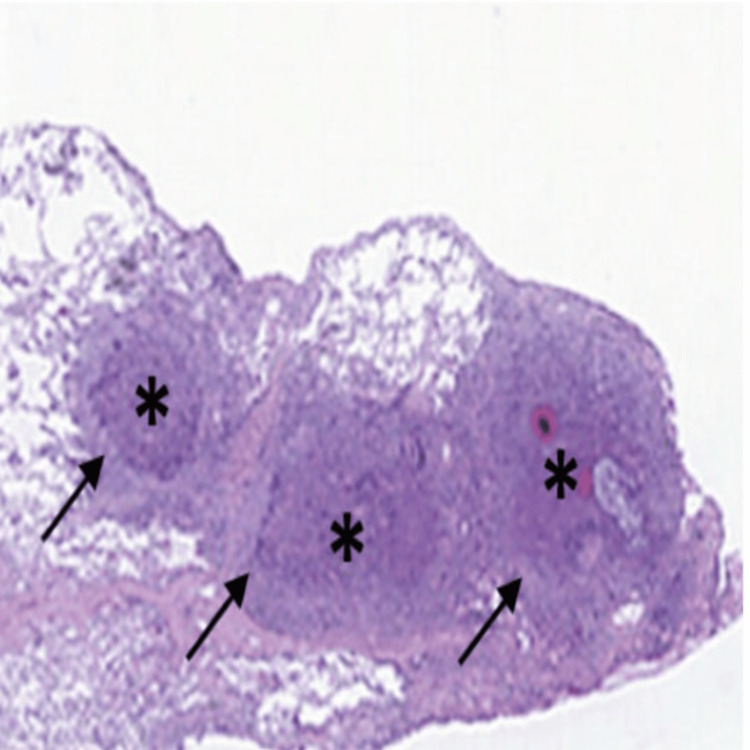
Hematoxylin and eosin-stained transbronchial biopsy specimen (magnification ×100) showing a discrete granuloma (black arrows) with a central bacterial mass (black asterisk) surrounded by neutrophils

**Figure 3:**
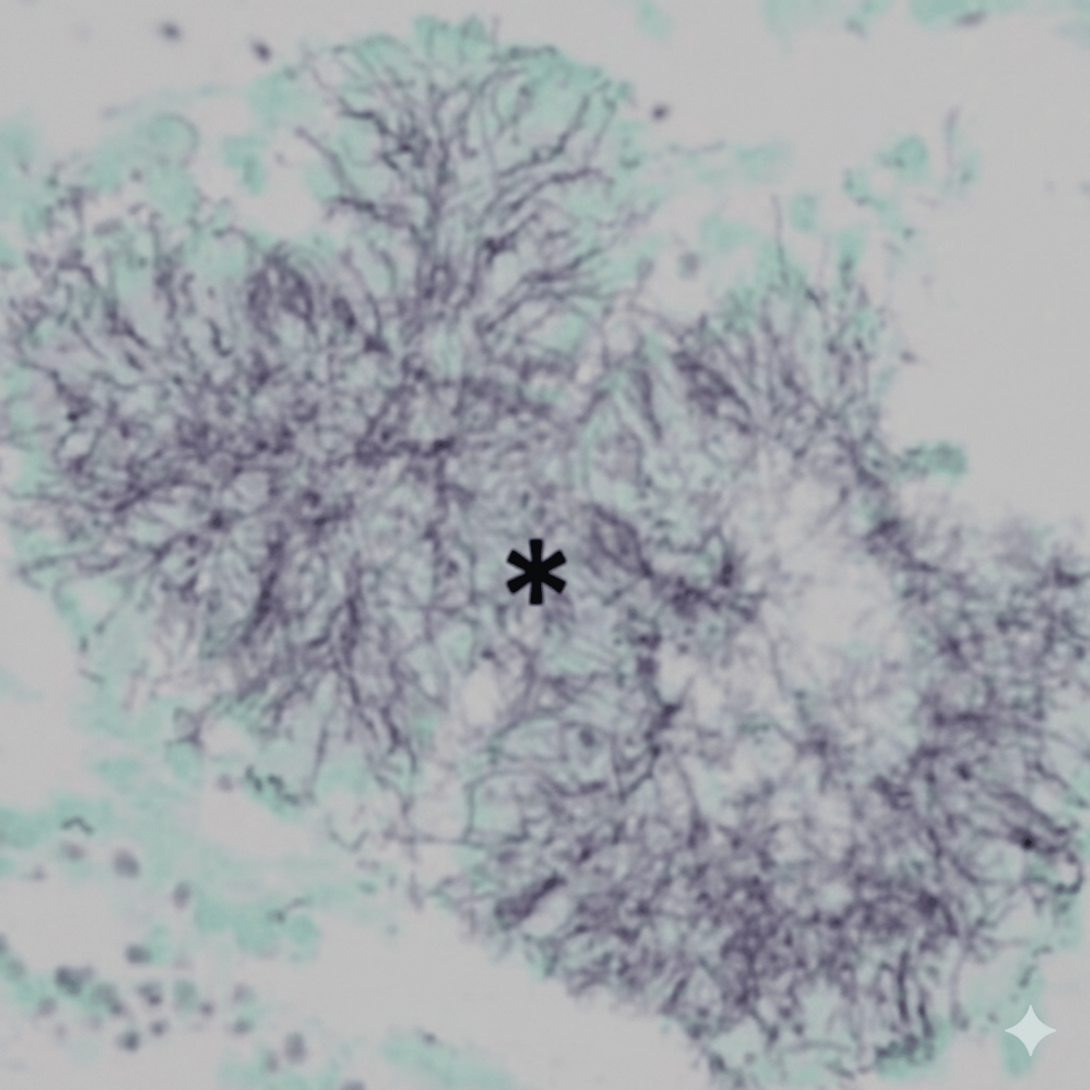
Grocott-Gomori’s methenamine silver-stained transbronchial biopsy specimen (×400) showing filamentous bacteria arranged in a radiating pattern (black asterisk) consistent with sulphur granules

**Figure 4:**
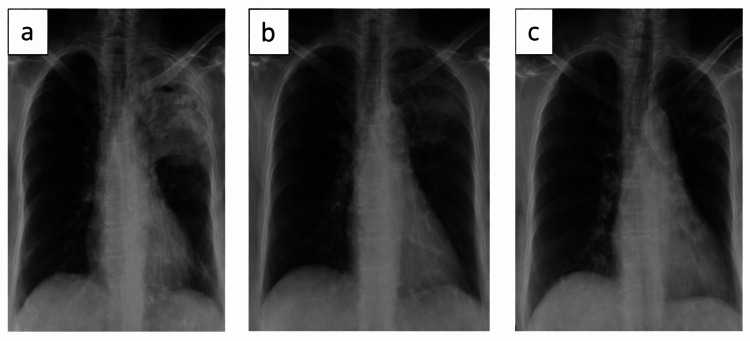
Chest roentgenogram at baseline (a), two months (b), and at the end of actinomycosis treatment (c)

A 63-year-old lady with type 2 diabetes mellitus presented with low-grade fever, weight loss, and dry cough of two months’ duration. The initial chest radiograph showed left upper zone opacity. Sputum induced with hypertonic saline was of poor quality, and the Xpert/MTB assay of the sample was negative. She was empirically started on ATT, as she denied diagnostic bronchoscopy. She remained symptomatic despite being on weight-based ATT. A high-resolution computed tomography of the chest done at presentation to our hospital showed persistent consolidation with cavitation in the left upper lobe (Figure 1). Laboratory investigations at initial evaluation showed leukocytosis of 12×10^9^/L (normal range: 4-11×10^9^/L), elevated C-reactive protein (CRP) of 12 mg/L (normal range: <5 mg/L), deranged fasting blood glucose of 140 mg/dL (normal range: 70-99 mg/dL), and deranged HbA1C of 9% (normal range: <5.7%); serum electrolytes, serum creatinine, urine routine, and microscopy were within normal limits. To confirm diagnosis and evaluate causes of treatment failure, such as drug resistance, secondary infection, or malignancy, bronchoscopy with bronchioalveolar lavage (BAL) and transbronchial lung biopsy (TBLB) was performed. The BAL sample yielded negative results for acid-fast bacilli, fungi, aerobic bacteria, and the Xpert/MTb assay. Histopathological analysis of the TBLB specimen demonstrated discrete granulomas with central bacterial colonies surrounded by neutrophils (Figure 2). Grocott-Gomori’s methenamine silver nitrate stain highlighted filamentous bacteria arranged in a radiating fashion consistent with sulphur granules (Figure 3). The patient was started on intravenous meropenem with a provisional diagnosis of actinomycosis. Anaerobic culture of preserved BAL sample showed growth of *Actinomyces israelii* on the seventh day of incubation, with sensitivity to amoxicillin, clindamycin, and doxycycline. Based on the susceptibility report, the antibiotic was deescalated to oral amoxicillin-clavulanate. The patient’s ATT was stopped, and she received a total of 10 months of actinomyces-specific treatment, resulting in marked clinical and radiological resolution. Follow-up imaging showed near-complete clearance of the left upper lobe opacity (Figures 4a-4c).

## Discussion

Pulmonary actinomycosis is an uncommon chronic suppurative and granulomatous infection, accounting for 20% of the total burden of actinomycosis. Underlying immunocompromised states, such as diabetes mellitus, are a major risk factor in the pathogenesis of this infection [3]. Diagnosis of pulmonary actinomycosis is often missed or delayed owing to nonspecific clinical signs and radiological features [4]. Pulmonary actinomycosis can be easily overlooked as a cause of pneumonia in regions with high PTB prevalence. Histopathological examination and anaerobic culture are pivotal to achieve accurate diagnosis [2]. Treatment depends on the administration of sensitivity-based antibiotics, with the duration of therapy guided by the severity of the disease. The presence of necrosis or cavitation (as in our case), presence of fistula or sinus, empyema, and bony involvement is defined as severe disease requiring 6-12 months of therapy. Although indolent, pulmonary actinomycosis can result in significant morbidity and even fatality if left unattended [5]. Our case highlights the misleading patterns of actinomycosis, the role of histopathology and anaerobic culture, and the perils of missed diagnosis.

## Conclusions

In conclusion, clinicians should keep the possibility of uncommon infections, such as actinomycosis, in patients with suspected PTB with a poor response to standard therapy. Timely diagnosis and targeted antimicrobial treatment can reduce morbidity and avert subsequent surgical interventions.
